# Significance or Scatter—Statistical Evaluation of Rapid Chloride Migration Test Results for Sprayable Cement Mortar

**DOI:** 10.3390/ma15176050

**Published:** 2022-09-01

**Authors:** Stefan Ullmann, Sven Nordsiek, Dirk Lowke

**Affiliations:** Institute of Building Materials, Concrete Construction and Fire Safety (iBMB), Technische Universität Braunschweig, 38106 Braunschweig, Germany

**Keywords:** rapid chloride migration test, *t*-test, coefficient of variation, repair mortar

## Abstract

In order to evaluate the influence of certain experimental or material-related conditions on results of the rapid chloride migration test (RCM test), statistical tests within and between samples are necessary. Thus, it needs to be clear which scatter a sample or a population of results is already subjected to without external influences due to the test method itself. So far, however, literature values for the appropriate statistical variable (coefficient of variation, CoV) explicitly valid for mortar or fine-grained concrete, e.g., for concrete repair, are missing. Therefore, we suggest a specific mortar CoV based on our own results of RCM tests performed on a cement-rich, sprayable mortar based on ordinary Portland cement. For the evaluation of external influences on a sample in comparison to a reference sample, we developed a significance criterion based on a statistical hypothesis test. The sensitivity and the reliability of this criterion is demonstrated on various results from RCM tests on mortar specimens, according to the test specifications and with deliberately chosen deviations from it. In addition, we point out the parameters included in the calculation of the rapid chloride migration coefficient that are the most sensitive to unintentional errors.

## 1. Introduction

### 1.1. The Chloride Migration Coefficient as Key Parameter

The object of investigation in building materials science is often about a certain effect of an investigated aspect on a specific material property characterised by a standardised testing method. To interpret the results, they are usually compared to test results from a reference sample, e.g., OPC specimens.

However, in order to be able to actually determine or evaluate the effect of an aspect on a test result (referred to as “significance” in the following), the amount of scatter of the test method itself is relevant. In addition, it is important to know how robust the test is to unintentional inaccuracies (e.g., due to a tester’s influence), and how large these resulting deviations in the test results are. Thus, reliable statistical tools for the evaluation of test results are of great importance. Otherwise, the researcher could incorrectly evaluate test results in the scatter range of the method as a significance. Therefore, statistical evaluation variables and associated limit values have to be defined.

The non-steady-state chloride migration coefficient D_nssm_ is the most important material parameter for the service life design of cementitious materials such as concrete or mortar. It is determined in the rapid chloride migration test (RCM test) in the laboratory. At the international level, the test specification valid for the implementation is NT Build 492 [1]. It specifies all necessary boundary conditions to conduct the test (e.g., casting and curing of the specimens, the technical settings for the test itself) and assures comparability of the test results. Any deviation from the recommended settings will lead to results that are not comparable.

### 1.2. Existing and Missing Criteria for the Statistical Evaluation of the Chloride Migration Coefficient

A basic statistical parameter to evaluate scattering of the non-steady-state chloride migration coefficient is the coefficient of variation (CoV), defined as the quotient of the standard deviation *s* and the mean value $\bar{x}$ of a sample:

(1) $$\mathrm{CoV}\;=\;s/\bar{x}$$

The international test specification NT Build as well as the German test specification for the implementation of the RCM test (BAW leaflet MDCC [2]) contain specific CoV limit values for concrete specimens based on OPC, mostly originating from the work of Tang [3] and Gehlen [4], see Table 1. Furthermore, a distinction between the CoV of repeatability and the CoV of reproducibility is necessary. The CoV of repeatability is the sharper criterion that is valid for test results determined by the same person in the same laboratory, with the same experimental set-up over a short period. If various researchers, at different locations and/or with different instruments conduct the tests, the weaker CoV of reproducibility is used [5].

The test specifications state validity for cementitious materials without a differentiation between concrete and mortar. Since the investigations that led to the listed CoV essentially go back on concrete specimens, it is worth questioning whether they are also valid for specimens with finer aggregates, such as mortar. This aspect becomes increasingly relevant as these materials are widely used in terms of concrete repair and in 3D printing. In both cases, the importance of durability aspects (and as a result the evaluation of the D_nssm_-value) is growing [6,7].

Above all, the most important and critical aspect regarding the CoV and the evaluation of test results is that it is only suitable for the evaluation of scattering within on population or sample. The CoV is not suitable to evaluate significant deviations of one sample in comparison to a reference sample or value. That is necessary as soon as the effect of a certain experimental or material-related boundary condition on the chloride migration coefficient is investigated.

Therefore, we developed a multistage criterion for the evaluation of significance of a tested aspect on the chloride migration coefficient. The investigations go back on our test results from specimens of a sprayable mortar with high cement content for 3D printing or concrete repair. The results also lead to a proposal for a sharper CoV to detect deviations from the standard test routine for mortar. By intentionally deviating from the test routine described in the specifications, we investigate the robustness of the RCM test.

In addition, we present an in-house developed software, which enables the digital determination and the statistical evaluation of the shape of the front of chloride ingress and the related steadiness of the chloride penetration depth.

## 2. Materials and Methods

### 2.1. Materials and Specimen Preparation

Based on the formulation of a sprayable cement mortar used for the Shotcrete 3D Printing (SC3DP) [8], we composed a material with a w/c ratio of 0.45 and 0.3% b.w.o.c. of superplasticizer (Table 2).

According to NT Build 492, we casted the test specimens in a cylinder with a diameter of 100 mm and a height of 200 mm and extracted a slice of 50 mm thickness from the middle section. We cured the specimens for 28 days at a temperature of 20 °C under water until the day of testing.

### 2.2. Rapid Chloride Migration Test and Theoretical Background

We designed the experimental set-up for the rapid chloride migration test (RCM test) according to NT Build using a catholyte reservoir box capable of containing up to six specimens at the same time.

The test duration and the applicable voltage were determined based on the initial current measured at 30 V according to Table 1 of NT Build. As we worked with a binder-rich system, we multiplied the value for the initial current (90–120 mA) with a factor of 0.5, as recommended by NT Build. Thus, the reference tests were conducted for 24 h and at a voltage of 30 V.

After the test, we split the specimens and applied the indicator (0.1 M silver nitrate solution) to the fracture surface. The chloride-containing area became visible in a light grey colour, whereas the area without chloride turned brown. According to NT Build we determined the average value of the penetration depth x_d_ from nine points equally distributed along the cross-section.

For the standard testing procedure with a catholyte solution of 10% NaCl, the non-steady-state chloride migration coefficient D_nssm_ can be calculated using x_d_ and Equation (2).

(2) $$D_{\mathrm{nssm}}=\frac{0.0239\;\left(273+T\right)\;L}{\left(U-2\right)\;t}\;\left[{\;x}_{d}-0.0238\;\sqrt{\;\frac{\left(273+T\right){\;L\;x}_{d}}{U-2}}\;\right]$$

where:

D_nssm_: non-steady-state chloride migration coefficient [10^−12^ m^2^/s]

U: absolute value of applied voltage [V]

T: average temperature of the anolyte solution [°C]

L: thickness of the specimen [m]

x_d_: average value of the penetration depth [m]

t: test duration [h]

### 2.3. Variations in Specimen Preparation and Testing Procedure

In our investigations of the experimental influences on the chloride migration coefficient, we were mainly interested in two aspects:

- effects of the preparation of the specimens and;
- effects of the testing procedure.

In order to point out possible effects of the specimen preparation on the chloride migration coefficient we additionally prepared specimens with slight modifications to the recommendations in NT Build regarding the extraction of the specimens and simplified handling.

The variations in the test procedure are in the duration of the test, the applied voltage and the concentration of the catholyte solution. We labelled the specimens systematically according to the respective test parameters (see Table 3). For example, a test result labelled 3/30/24/10 refers to a single test result of one of the three specimens extracted from a cylinder and tested at 30 V for 24 h in a 10% NaCl solution.

As we changed the concentration of the solution, we had to adjust the equation as instructed by NT Build. Equation (3) implicates the inversed error function [9] in dependency on the concentration (c_0_) of NaCl in the solution, see Equation (3). The calculated values for this term are listed in Table 4. The change of the equation can be traced using the NT Build.

(3) $${\mathrm{erf}}^{-1}\;\left(1-\frac{2c_{d}}{c_{0}}\right),$$

where:

erf^−1^: inverse of Gaussian error function

c_d_: chloride concentration at which the colour changes = 0.07 N for OPC

c_0_: chloride concentration in the catholyte solution (variable).

### 2.4. Analysis of the Test Results

The average penetration depth x_d_ is the mean of the thickness values of the chloride-affected region measured on the split specimen at predefined intervals. According to NT Build, x_d_ is defined as the mean measured at nine equidistant intervals, where a space of 10 mm at each side is excluded to avoid any edge effects.

**Figure 1:**
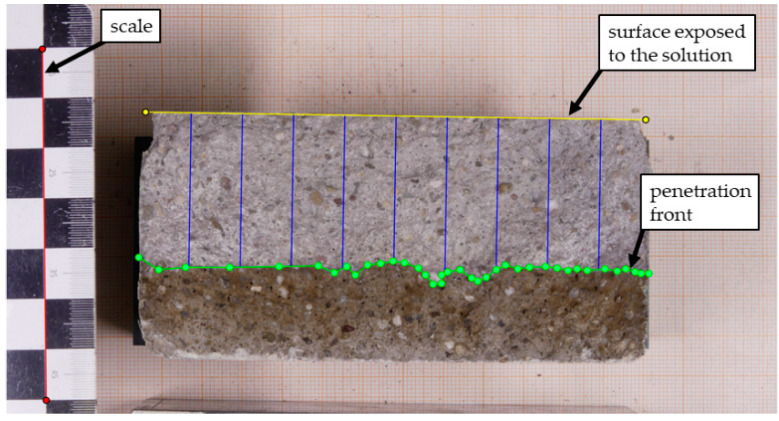
Digital image of a specimen after processing with the RCM app to compute the mean penetration depth x_d_. The blue lines indicate the positions where the thickness of the chloride-containing layer has been determined.

Usually, the thickness measurements are performed with a Vernier caliper. In our study, we determine the penetration depths by taking a digital photo perpendicular to the fracture surface. Processing the digital image with a software tool (RCM-App) allows magnification and subsequent contrast improvement, if necessary, to support a clear distinction between the section of the specimen containing chloride ions and the unaffected part. For the conversion between distances in the image and actual distances, we include a scale in each photo placed within the plane of the fracture surface (see Figure 1).

After marking both the specimen’s surface exposed to the solution and the penetration front as lines in the digital image, the penetration depth can be determined automatically at any intervals according to the chosen testing procedure. In addition to the recommended 9 points, we decided to analyse 101 points with the RCM app. Finally, we characterise the resulting distribution of penetration depths by computing its minimum and maximum values as well as median, mean and standard deviation of the distribution.

### 2.5. Statistical Analysis of Significance

For the statistical analysis of the test results, we chose the *t*-test according to Gosset [10]. The *t*-test is a tool for the statistical hypothesis testing that is particularly suitable for small samples (n < 30). It proofs a certain hypothesis wrong or right.

The statistical variables required for the test are the mean value $\bar{x}$, the standard deviation s or the variance s^2^ and the number of results n. It is tested whether the mean of two samples or the mean of one sample and a single reference value differ significantly or incidentally.

In this paper, we used the two-sample *t*-test as we did not refer to a single value but to the mean of a reference sample. The one-sample *t*-test is useful to evaluate the mean of a sample in reference e.g., to a standardised limit value.

The following requirements must be met to run a *t*-test [10]:

- the samples are chosen randomly;
- the samples do not depend on each other;
- the tested values are on the same parametric scale;
- the results are distributed normally;
- homogeneity of variance for the two samples.

The homogeneity of variance has to be tested with the so called Levene’s test or F-test [10]. The variance of two samples is considered homogeneous if

(4) $$F\;=\frac{s_{A}^{2}}{s_{B}^{2}}<{\;F}_{\mathrm{crit}}$$

where:

F: F-value (statistical variable)

s_A_^2^: variance of sample A (the higher variance needs to be the numerator)

s_B_^2^: variance of sample B

F_crit_: critical F-value according to tables [10].

If the Levene’s-test confirms homogeneity, the two-sample *t*-test is performed with equal (5a) and unequal variances (5b), respectively:

(5a) $$t=\frac{\left|{\bar{x}}_{A}-{\bar{x}}_{B}\right|}{\sqrt{\left(\frac{\left(n_{A}+n_{B}\right)}{n_{A\;}n_{B}}\right)\;\frac{\left(n_{A}-1\right){\;s}_{A}^{2}+\left(n_{B}-1\right){\;s}_{B}^{2}}{n_{A\;}+{\;n}_{B}-2}}\;}<{\;t}_{\mathrm{crit}}$$

(5b) $$t=\frac{\left|{\bar{x}}_{A}-{\bar{x}}_{B}\right|}{\sqrt{\frac{s_{A}^{2}}{n_{A}}+\frac{s_{B}^{2}}{n_{B}}}}<{\;t}_{\mathrm{crit}}$$

where:

t: t-value (statistical variable)

$\bar{x}$_A_: mean value of sample A

$\bar{x}$_B_: mean value of sample B

n_A_: number of values of sample A

n_B_: number of values of sample B

s_A_^2^: variance of sample A

s_B_^2^: variance of sample B

t_crit_: critical t-value according to tables

The critical t-value t_crit_ is dependent on the degree of freedom *df* and is listed in generally valid tables [10]. The t_crit_-value varies because there are fewer degrees of freedom in the *t*-test with unequal than with equal variances and because it depends on the method of the test (one-tail (right or left) or two-tail, see below). The value is usually chosen for a confidence level of 95%.

For the evaluation of the mean values of the test results (significance testing), an initial hypothesis H_0_ and an alternative hypothesis H_1_ need to be formulated. If the t-value is below the critical value t_crit_, H_0_ is proven right. Otherwise, H_0_ is proven wrong, and H_1_ becomes relevant.

In the context of this paper, H_0_ has to be:

**Hypothesis 0** **(H_0_)**. *The investigated aspect does not significantly affect the test result.*

The formulation of the alternative hypothesis H_1_ is possible in three ways. It determines the method of the test and thus the critical t-value:

1. *“the investigated aspect increases the test results significantly”*—right tail test;
2. *“the investigated aspect decreases the test results significantly”*—left tail test;
3. *“the investigated aspect affects the test results significantly”*–two-tailed test.

The correct formulation of H_1_ is of high importance for the evaluation of the test results.

### 2.6. Multistage Criterion to Statistically Evaluate Significance or Scatter

**Figure 2:**
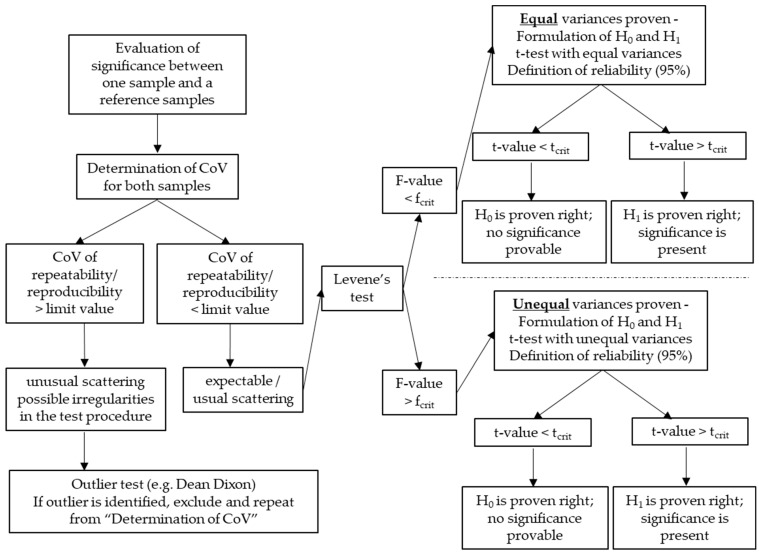
Proposed multistage criterion to evaluate significance or scatter of single samples or two samples of chloride migration coefficients.

Considering the presented aspects regarding the CoV and the two-sample *t*-test, it becomes obvious that a multistage criterion where the tests are set in line is necessary to correctly evaluate significance or scatter. The proposed approach is visualised in Figure 2. Comparable approaches are suggested in the field of non-destructive in situ strength assessment of concrete for the identification of test regions for the evaluation of non-destructive testing presented in [11].

## 3. Results

### 3.1. Effect of the Specimen Preparation and the Test Procedure

Table 5 shows the results of the variations related to the specimen preparation, i.e., the extraction of one or three specimens from a cylinder. The data refer to a certain number of results n obtained by the same researcher under the same conditions. Thus, the calculated CoV reflects the CoV of repeatability. The limit value according to NT Build is 9%. We analysed the test results according to the introduced multistage criterion in Figure 2. H_0_ is *“the sample preparation does not affect the test results”*, H_1_ in this case is *“the preparation affects the test results”* as it is not clear whether it increases or decreases the D_nssm_-value. Thus, we did a two-tailed *t*-test with a reliability of 95%.

We derived three conclusions from the results above:

- the CoV of repeatability of the investigated repair mortar is with 6.6–6.9% lower than the known values for concrete, see Table 1;
- the effect of the preparation of the specimen, i.e., the number of samples taken from a cylinder, on the chloride migration coefficient is negligible (t < t_crit_);
- in terms of effectiveness, it is therefore reasonable to extract 3 specimens from one cylinder, instead of 1 specimen per cylinder as recommended by NT Build.

The results of variations related to the conduction of the test are shown in Table 6. The chloride migration coefficient was determined on three samples taken from each cylinder, i.e., according to our modified specimen preparation procedure. The variations in the test procedure include the applied voltage, the test duration and the concentration of NaCl in the solution. The hypotheses H_0_ and H_1_ were the same as mentioned before, as again the test result could be affected both ways. Thus, we ran a two-tailed *t*-test with equal and unequal variances and with a reliability of 95%.

The multistage criterion was developed especially for these small numbers of test results. Uncertainties are covered by the critical values related to the degrees of freedom.

It is noticeable, that all of the coefficients of variation are well below 9% and, with few exceptions, in the same range of 2–5%. That is also expressed in the results of the Levene’s test, that proved equal variances for all samples except from 3/30/24/15. This sample showed a very low CoV of 0.2% only, which is a significant difference to the reference sample.

The two-tailed *t*-test pointed out significant differences from the reference sample for four samples (see Table 6, last column) that can be attributed to:

- low voltage during the test;
- high voltage in combination with short test duration;
- low concentration of NaCl in the catholyte solution.

Regarding the last point, it is worth noting, that the t-value for both samples with differing NaCl concentration (5% and 15%) was near the t_crit_-value. Whereas the t-value for 5% was slightly above t_crit_, the t-value of 15% was slightly below t_crit_. Thus, a change in the NaCl concentration by ±5% should be considered as a parameter with a significant effect on the chloride migration coefficient.

### 3.2. Sensitivity of Parameters and Influence of the Tester

In addition to the scattering of RCM test results that are caused by inhomogeneities of the tested concrete or mortar, the tester’s influence on the test result has to be considered. As this has a direct effect on the scattering within a single sample, the maximum permissible influence of the tester is defined by the limit value of the CoV. Thus, accuracy is indispensable, especially as the proposed multistage criterion is otherwise not applicable.

**Figure 3:**
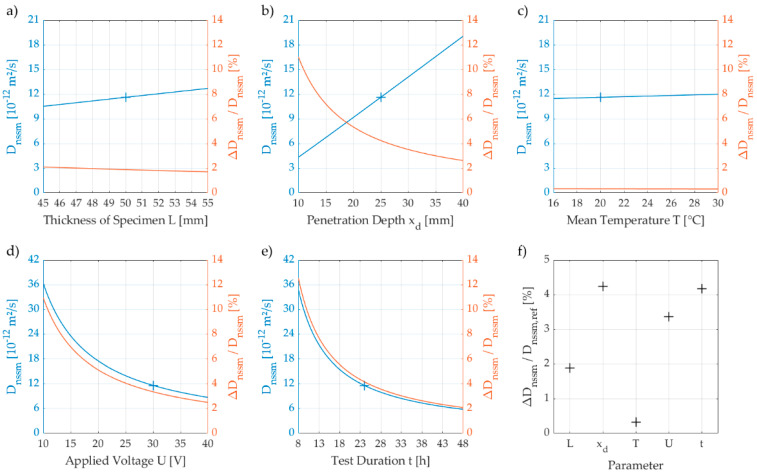
Simplified sensitivity analysis of the non-steady-state chloride migration coefficient D_nssm_ to variations in the thickness of specimen **a**), the penetration depth **b**), the mean temperature of the solution **c**), the applied voltage **d**) and the duration of testing **e**). Blue colour refers to the measured D_nssm_-values, red colour to the relative deviation Δ_Dnssm_/D_nssm_, that illustrates the impact of small variations (1 part per unit) of each parameter on the D_nssm_-value. The crosses indicate the reference value D_nssm,ref_. Panel **f**) displays the relative deviations observed for the reference parameters.

To reduce scatter and avoid high CoV, the tester needs to be sensitised to the working steps where unintentional errors have the greatest impact on the test result. Therefore, we performed a simplified sensitivity analysis and quantified the effects of each parameter of equation (2) on the D_nssm_-value, see Figure 3. As reference, which is indicated with crosses in Figure 3a)–e), we chose a specimen with a thickness of 50 mm (L) and a penetration depth of 25 mm (x_d_). The boundary conditions for the reference were set to 30 V (U), 24 h (t) and 20 °C (T).

The graphs in panels a)–c) indicate a linear relationship between the increase of the D_nssm_-value and the thickness of the specimen (L), the penetration depth (x_d_) and the temperature of the solution (T). Furthermore, the graphs in panels d) and e) show a non-linear decrease of the D_nssm_-value with increasing voltage (U) and duration of the test (t), respectively.

In addition to general relationships between the D_nssm_-value and the experimental parameters, we considered the effect of small variations of single parameters (resulting from observation errors) on the D_nssm_-value. We determined the difference Δ D_nssm_ between the maximum and the minimum of the D_nssm_-value within an interval of one unit for each parameter and normalised it by the D_nssm_-value attributed to the mean of the interval. As illustrated by the graph in panel f), the impact of an error related to the documentation of the temperature is much smaller than an error related to the power supply during the test.

We identified the penetration depth as the most sensitive variable as an error of 1 mm during the analysis of the front of ingress results in an error of more than 4% in the chloride migration coefficients. This effect intensifies the smaller the actual penetration depths are (up to 11% per mm at a mean penetration depth of 10 mm in contrast to 3% per mm at a mean penetration depth of 40 mm). This observation is in line with the recommendations regarding the penetration depths to be aimed for in RCM tests on mortar specimens given by Spiesz et al. [12].

### 3.3. Effect of the Penetration Depth Determination Procedure

After the identification of the penetration depth to be the most sensitive parameter to errors in the determination of the non-steady-state chloride migration coefficient we ran a further study on the potential of minimising the tester’s influence by using the RCM app that we developed for the analysis of unsteady penetration fronts.

The study focused on three aspects:

1. Are there deviations between an analysis with the RCM app and a measurement using a caliper?
2. Does the number of points of depth measurement affect the mean penetration depth? Here, we have compared the mean value of the penetration depth determined at 9 points according to NT Build with a mean value derived from 101 points.
3. Is the result determined with the RCM app reproducible for different users?

**Figure 4:**
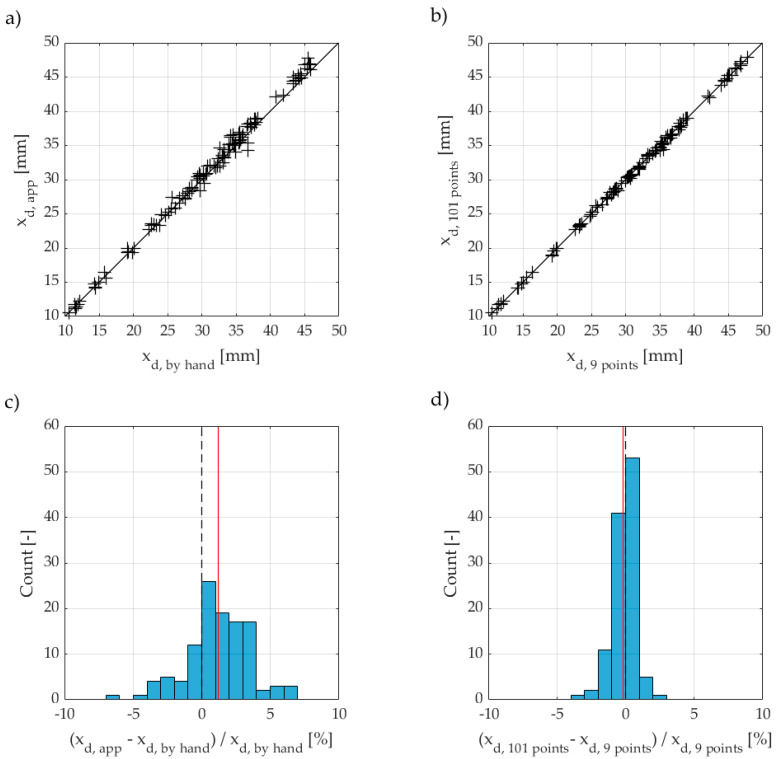
Direct comparison of the penetration depths x_d_ determined with the RCM app or a caliper (**a**) at 101 or 9 points with the app (**b**) and relative comparison of the results (**c**,**d**). The mean deviation of the relative results is indicated by a vertical red line.

We summarised the results for the study on aspects 1 and 2 in Figure 4. The data refers to 57 specimens, i.e., 114 halves.

The distribution of the relative deviations indicates slightly larger values for the penetration depth determined with the app than for a measurement using a caliper, see Figure 4a. The presented data show that the mean deviation between both methods is +1.2%, with maximum deviations of −6.8% and +6.7%, respectively (see Figure 4d, vertical red line). We were able to trace back both extremes to specimens that broke crookedly during splitting.

Surprisingly, the results from the analysis of 101 points do not differ significantly from the analysis of 9 points. Although there are single results with deviations up to −3.1% and +2.6%, respectively, the mean of the distribution shows a negligible deviation of −0.2% (see Figure 4d, vertical red line).

To investigate the sensitivity of the RCM app to the influence of different users (aspect 3), twenty specimens were analysed independently by two researchers. The results were compared for the analysis of 9 and of 101 points. Table 7 shows the statistical values of the analysis. For the calculation of the mean deviation and the standard deviation we transferred the deviations to absolute values.

From Table 7, we see that the minimal and the maximal deviation is about the same whether it is between 9 and 101 points or between min/max. That is also expressed in the mean deviation that we calculated from the absolute values of the deviations.

## 4. Discussion

### 4.1. Multistage Criterion to Evaluate Significance or Scatter of D_nssm_-Values

The introduced multistage criterion (see Section 2.6) enables a targeted identification of significant deviations of one sample from a reference sample. In particular, the CoV at short test durations in combination with high voltage differed significantly from the reference test procedure (see Section 3.1, Table 6). Based on this result, we advise against deliberately shortening the test duration by applying higher voltages in order to test more specimens in a shorter time. In order to increase the depth of ingress in mortars with high density, it is more accurate to prolong the test duration at a moderate voltage than to increase the applied voltage and shorten the test duration.

The significance criterion also identified the sample tested at low voltage. This can be explained with the resulting lower penetration depth compared to the reference sample. Spiesz et al. made corresponding observations as they detected high CoV from samples with low penetration depth [12].

A change in the NaCl concentration by ±5% significantly affects the chloride migration coefficient. However, minor deviation in dosage in a range of 1–2% should not have any effect on the test results, but should disappear in the expected scatter of the test.

The first stage of the presented criterion includes a limit value for the CoV of each sample. For the determination of a limit value, we refer to the existing limit values given by the test specifications (9% according to NT Build). Our investigations indicated that this CoV could also be used for mortar even if the limit value was derived from mainly concrete samples. In the light of our results, however, it appears as a conservative limit, as the penetration front in mortar specimens in general appears much smoother due to the finer aggregates than in concrete specimens. Therefore, we propose to use a CoV of 7% as guidance for evaluating the scattering of chloride migration coefficients determined on mortar samples.

### 4.2. Minimisation of the Tester’s Influence

The analysis of the experimental parameters determining the chloride migration coefficient in Section 3.2 revealed, that the evaluation of the penetration depths is the greatest source of error in the calculation of the D_nssm_-value. The use of a software tool comparable to the introduced RCM app (see Section 3.3) appears helpful as it avoids misreading or misalignment of the caliper during the measurement and preserves the analysis of the penetration depth in a visualisation on the photo of the specimen.

The possibility to analyse arbitrary numbers of penetration depths helps to evaluate the penetration depths (and the D_nssm_-value) for specimens with unsteady penetration fronts (e.g., due to cracks or large aggregates). However, the investigation also showed that the evaluation of only 9 points for specimens with uniform penetration fronts already leads to a satisfying accuracy.

Still, the analysis with the RCM app caused a certain error and constantly overestimated the D_nssm_-value compared to the measurement using a caliper. Therefore, we identified some possible reasons:

- image distortion;
- height difference between the scale and the fracture surface;
- fracture surfaces that are uneven in the direction of the image plane.

Whereas the first two influencing factors are avoidable with careful handling, the third one is unavoidable as the image lacks the third dimension (depth information). It can only be minimised by a deliberate positioning of the line that marks the surface exposed to chloride (yellow line in Figure 1).

## 5. Conclusions

The aim of the investigations presented here was to provide a standardised procedure for the evaluation of specific experimental or material-related influencing parameters on the results of the rapid chloride migration test. The tolerable coefficient of variation given in the test specifications is suitable to evaluate the scatter within one sample (group of results determined under the same conditions), but not to compare one sample with a reference sample.

As both the scatter within one and between two samples are important for a comprehensive evaluation of test data, we developed a multistage criterion, including the determination of the CoV for mortar, followed by the Levene’s test and the *t*-test as tools for statistical significance testing.

From the investigations, we can draw the following conclusions:

- The maximum CoV of 9% for concrete given in the test specifications, i.e., 9%, is adoptable for mortar. However, it appears that a lower CoV of 7% might be more suitable to evaluate test results from mortar specimens. The reason for this is the smaller aggregate size compared to concrete, which results in a more uniform penetration front and thus to less scatter in the test results.
- The analysis of the test results using the criterion showed that it is possible to precisely determine effects (variations in sample preparation) that have a significant impact on the test results, and to differentiate the significant variations from those that just led to expectable scattering. Although we tested mortar specimens, the criterion is valid for concrete and mortar as it is has a statistical background that is not dependent on the tested material.
- In a study of the sensitivity of experimental parameters, we identified the evaluation of the penetration depth as the most influential parameter for the calculation of the chloride migration coefficient. In this context, we have shown that the use of an image analysis software enables a digital and flexible determination of the penetration depth and reduces the risk of incorrect manual measurements by a tester.

## Figures and Tables

**Table 1:** CoV of repeatability and of reproducibility suggested by certain test specifications to evaluate scatter of D_nssm_-values for OPC-concrete specimens.

Test Specification	CoV of Repeatability	CoV of Reproducibility
NT Build	9%	13%
Baw Leaflet MDCC	11%	20%

**Table 2:** Mixture proportion of the sprayable cement mortar.

Material	Proportion [kg/dm^3^]
Sand (max. particle size: 3.15 mm)	1255
Limestone powder	100
Cement (CEM I 52.5 N)	600
Water	270

**Table 3:** Labelling of the test modifications.

No.	Label	Description
1	1, 3	number of specimens extracted from a cylinder ^1^
2	20, 25, 30, 35, 40	applied voltage (in volts) during the test
3	8, 24, 48	duration of the test (in hours)
4	5, 10, 15	NaCl concentration in the catholyte solution (in percent)

^1^ We simplified the handling as we alternatively extracted three disks with a height of 50 mm from a cylinder according to NT build 492, starting 10 mm underneath the former upper side of the cylinder. Each of the three disks leads to a single test result.

**Table 4:** Results from Equation (3) for varying chloride concentrations c_0_ in the solution.

Percentage of NaCl	c_0_ [N]	1 − $\frac{2c_{d}}{c_{0}}$	${\mathrm{erf}}^{-1}\left(1-\frac{2c_{d}}{c_{0}}\right)$
5% 10%	0.95 ≈2.0 ^1^	0.85 0.93	1.03 1.28
15%	2.85	0.95	1.39

^1^ according to NT Build. More accurate would be 1.9 N.

**Table 5:** Comparison of D_nssm_ determined from specimen preparation according to NT Build (1/30/24/10) and from modified specimen preparation 3/30/24/10.

Statistical Value	1/30/24/10 (acc. NT Build)	3/30/24/10
Number of results (n)	15	16
Mean value $\bar{x}$ [10^−12^ m^2^/s]	11.7	12.1
Standard deviation s [10^−12^ m^2^/s]	0.81	0.79
Coefficient of variation (CoV)	6.9%	6.6%
F-value/F_crit_	1.085 < 2.329—equal variances	
t-value/t_crit_	0.553 < 2.040—H_0_ is proven right	

**Table 6:** Comparison of D_nssm_ determined with variations in the applied voltage, the test duration and the concentration of NaCl in the solution in reference to 3/30/24/10.

Variation	Mean D_nssm_^1^	CoV	<9%	Variances (Levene’s Test)		Significance (*t*-Test)	
				Equal	Unequal	No (H_0_)	Yes (H_1_)
3/**20**/24/10	14.2	4.3%	✓	✓			✓
3/**25**/24/10	13.0	2.5%	✓	✓		✓	
3/30/24/10	12.2	3.2%	✓	Reference test procedure			
3/**35**/24/10	13.1	5.0%	✓	✓		✓	
3/**40**/24/10	12.1	2.8%	✓	✓		✓	
3/**20**/**48**/10	12.2	6.7%	✓	✓		✓	
3/**25**/**48**/10	12.5	1.3%	✓	✓		✓	
3/**30**/**8**/10	15.4	2.4%	✓	✓			✓
3/**40**/**8**/10	15.5	3.4%	✓	✓			✓
3/30/24/**5**	13.5	3.0%	✓	✓			✓
3/30/24/**15**	13.3	0.2%	✓		✓	✓	

^1^ based on 3 D_nssm_-values.

**Table 7:** Statistical values [mm] related to the analysis of the tester’s influence on the test result.

Statistical Value	9 Points	101 Points
Number of results (n)	20	20
Minimal deviation [mm]	−0.77	−0.66
Maximal deviation [mm]	+0.51	+0.41
Mean deviation (abs. value) [mm]	0.26	0.24

## Data Availability

There is no accompanying data available.
